# Therapeutic Drug Monitoring for Precision Dosing of Janus Kinase Inhibitors: Protocol for a Prospective Observational Study

**DOI:** 10.2196/70312

**Published:** 2025-09-09

**Authors:** Jérémie Tachet, Laurent A Decosterd, Monia Guidi, François R Girardin

**Affiliations:** 1 Service of Clinical Pharmacology, Department of Medicine Lausanne University Hospital and University of Lausanne Lausanne Switzerland; 2 Laboratory of Clinical Pharmacology, Department of Laboratory Medicine and Pathology Lausanne University Hospital and University of Lausanne Lausanne Switzerland; 3 Centre for Research and Innovation in Clinical Pharmaceutical Sciences, Department of Education and Research Lausanne University Hospital and University of Lausanne Lausanne Switzerland; 4 Institute of Pharmaceutical Sciences of Western Switzerland University of Geneva, University of Lausanne Geneva, Lausanne Switzerland

**Keywords:** Janus kinase inhibitor, JAKI, abrocitinib, baricitinib, fedratinib, ruxolitinib, tofacitinib, upadacitinib, therapeutic drug monitoring, pharmacokinetics, population pharmacokinetic modeling

## Abstract

**Background:**

Janus kinase inhibitors (JAKIs) are small molecules used orally to treat inflammatory and hematological disorders. They have demonstrated impressive efficacy across multiple indications. However, concerns have emerged regarding their safety profile. Despite their growing clinical use, therapeutic drug monitoring is not yet established for JAKIs, but it could help address exposure-dependent efficacy and tolerability issues through individualized treatment approaches.

**Objective:**

This protocol aims to characterize the pharmacokinetics of the 6 most prescribed JAKIs in Switzerland (abrocitinib, baricitinib, fedratinib, ruxolitinib, tofacitinib, and upadacitinib) and identify factors influencing drug exposure. It seeks to explore exposure-response relationships to assess the impact of drug exposure markers on efficacy and safety. Ultimately, these findings will contribute to establishing therapeutic intervals.

**Methods:**

This prospective observational study, conducted throughout Switzerland, was approved by the Cantonal Ethics Committee in August 2023. Consenting adults (aged ≥18 years) who are capable of judgment and who are prescribed JAKIs are enrolled in the study, either for sparse sampling during routine medical visits to collect trough or random plasma concentrations or for a detailed pharmacokinetic substudy involving serial blood sampling over an 8-hour period. The characterization of JAKI pharmacokinetics, including associated variability and the effect of specific covariates, such as age, body weight, BMI, sex, disease type, drug-drug interactions, or concomitant pathophysiological conditions, will be performed using nonlinear mixed effect modeling techniques. Relationships between JAKI exposure and efficacy and safety will be assessed.

**Results:**

By August 2024, a total of 276 blood samples were collected from 107 patients, the majority being female individuals (n=62, 57.9%). The patients had a median age of 51 (range 17-87) years and a median body weight of 69 (range 39-132) kg. Most patients recruited were taking ruxolitinib (n=44, 41.1%), upadacitinib (n=39, 36.4%), or baricitinib (n=11, 10.3%).

**Conclusions:**

The framework of the study will allow the characterization of the pharmacokinetic profiles of JAKIs and their variability in real-world conditions. On the basis of novel therapeutic drug monitoring approaches, we expect to explore the relationship between drug exposure, treatment response, and tolerability, providing essential information for precise dose optimization.

**International Registered Report Identifier (IRRID):**

RR1-10.2196/70312

## Introduction

### Background

The substantial progress in managing inflammatory disorders has led to a broad range of therapies for patients with systemic autoimmune conditions. Biological agents, such as monoclonal antibodies, are the standard of care for many autoimmune disorders. However, they are characterized by variable pharmacokinetics and are associated with the production of autoantibodies over time. While biological agents achieve disease control in most patients, practical concerns remain regarding their stability and parenteral routes of administration [1].

Concurrently, the Janus kinase (JAK) and signal transducer and activator of transcription (STAT) signaling pathway plays a key role in cellular signal transduction involved in numerous acute and chronic inflammatory diseases, making the JAK/STAT target an alternative therapeutic approach for their treatment. JAKs are multidomain nonreceptor tyrosine kinases. The JAK protein family includes 4 isoforms (JAK1, JAK2, JAK3, and TYK2), while the STAT family consists of 7 proteins (STAT1, STAT2, STAT3, STAT4, STAT5a, STAT5b, and STAT6). The broad spectrum of possible combinations between JAK-STAT subunits specifically regulate cytokine signaling through class I and II receptors, resulting in pleiotropic effects on various immune and biological functions [2-4].

JAK inhibitors (JAKIs) are representatives of a novel class of small molecule immunosuppressants interfering with intracellular signaling triggered by the proinflammatory stimuli, such as cytokines [5]. JAKIs are increasingly used for various inflammatory conditions, such as immune-mediated arthropathies (eg, rheumatoid arthritis), immune-driven dermatological diseases, inflammatory bowel disease (IBD), myeloproliferative neoplasms (MPNs; eg, myelofibrosis, polycythemia vera), graft-versus-host disease (GvHD), and in some cases of myelodysplastic syndromes [4]. Compared to biological agents, JAKIs have a more rapid mode of action in acute situations and fewer issues associated with pharmacokinetics inertia [6-13]. Their immunosuppressant profiles are similar, but they offer the convenience of oral administration and lack of autoantibody formation. In Switzerland, the first JAKIs approved were ruxolitinib (2012) and tofacitinib (2013). Several other JAKIs have received marketing approval between 2020 and 2024.

Recently, safety concerns regarding JAKIs were reported in the literature. Across the class, common adverse drug reactions (ADRs) include infections; hematologic abnormalities; and laboratory changes, such as elevations in total cholesterol, low-density lipoprotein, liver transaminases, and creatine phosphokinase. Mycobacterial, fungal, viral, and other opportunistic infections, in particular herpes zoster reactivation, were associated with JAKIs [14-16]. In line with these findings, meta-analyses have reported a significant, dose-dependent increase in the risk of herpes zoster caused by reactivation of the varicella-zoster virus [17,18]. Hematological toxicities, including neutropenia, lymphopenia, anemia, and thrombocytopenia, are also dose-related ADRs and have been reported frequently with ruxolitinib and fedratinib [19-24]. Cardiotoxicity is a major safety concern, with reports of major adverse cardiovascular events, which include cardiovascular death, nonfatal myocardial infarction, and nonfatal stroke. The Oral Rheumatoid Arthritis Trial Surveillance study showed a higher risk of nonfatal myocardial infarction with tofacitinib compared to adalimumab in patients with rheumatoid arthritis aged 50 years or older with at least 1 cardiovascular risk factor [14]. In addition to thromboembolic events, such as deep vein thrombosis, pulmonary embolism, and venous thromboembolism, an increased risk of malignancies, including lymphoma, lung cancer, and nonmelanoma skin cancer, has also been reported [14,25]. These safety concerns emphasize the need for careful monitoring to ensure treatment efficacy while minimizing ADRs.

Therapeutic drug monitoring (TDM) involves measuring drug concentrations and adjusting the dosage to maintain exposure within a target range (ie, to optimize efficacy while limiting toxicity). Drug concentration exposure is the key factor for both drug efficacy and toxicity. However, drugs are mostly prescribed at standard dosage indicated by the manufacturer (“one-size fits all” approach), without accounting for interindividual pharmacokinetics variability. Multiple sources of variability can be identified, including demographic, environmental, clinical, and genetic factors. Besides this explained variability, a certain amount of pharmacokinetics variability remains unexplained, but deserves all the more to be taken into account in dosage individualization, which TDM makes possible. A priori recommended dosages vary sometimes according to the indication (eg, gastroenterologists use 2 to 3 times the dose of upadacitinib compared to rheumatologists) but are not individually adapted according to drug exposure. Holford and Sheiner [26] and Sheiner and Ludden [27] developed the population pharmacokinetics (popPK) approach to analyze TDM data, aiming to characterize and quantify variability, identify underlying sources, such as covariate effects, and simulate dosage adjustment. The popPK method ultimately allows for validating or proposing an alternative dosing regimen.

Drug concentration measurement has been increasingly used to optimize immunosuppressant treatments and is considered mandatory for the follow-up of organ transplantation. Typical drug candidates for TDM have clearly established exposure-response (in terms of efficacy and toxicity) relationships, a narrow therapeutic margin, limited intrasubject pharmacokinetics variability, significant interindividual pharmacokinetics variability, a lack of pharmacodynamic markers that reliably reflect therapeutic response or toxicity, and a sufficient treatment duration [28]. JAKIs fulfill several of these criteria, particularly the exposure-response relationship. An exposure-response relationship of JAKIs is well documented for most molecules regarding efficacy [29-41], while associations with toxicity have been reported but remain less consistently characterized across studies [29,32,37,42-45]. Despite relatively low intraindividual pharmacokinetics variability, JAKIs exhibit moderate to significant interindividual pharmacokinetics variability, susceptible to translating into insufficient efficacy in case of suboptimal concentration exposure, or unwanted toxicity in case of overexposure [32,43,46-50]. Several factors contribute to the variability in JAKI exposure, including their oral administration over long treatment periods, which may lead to suboptimal therapeutic adherence. As JAKIs are mainly metabolized by cytochrome P450 (CYP), they are subjected to many drug-drug interactions (DDIs) that may affect blood concentrations and, thus, exposures. Beyond DDIs, the inflammatory state itself may alter drug metabolism, a phenomenon known as phenoconversion, which can modify enzyme function and affect drug clearance [51-53]. On another note, baricitinib is primarily eliminated through renal filtration and secretion. Inflammatory-associated glomerular hyperfiltration may decrease its exposure; therefore, older patients with impaired renal function are at risk of accumulation [54]. Finally, inflammatory diseases and MPNs differ in pathophysiology, cytokine environment, and potential effects on drug metabolism. Higher concentrations of ruxolitinib were observed in patients with GvHD compared to patients with myelofibrosis under the same daily dose, due to a reduced clearance [43,55].

### This Study

We hypothesize that JAKIs could benefit from TDM to improve efficacy, safety, and efficiency according to the disease conditions. This exploratory study focuses on abrocitinib, baricitinib, fedratinib, ruxolitinib, tofacitinib, and upadacitinib and aims to investigate the pharmacokinetics characteristics of JAKIs in real-world patient populations. We aim to characterize pharmacokinetics profiles of JAKIs along with their variability based on measurements of circulating blood concentrations, patient characteristics, DDIs, pathophysiological status, and comedications using a popPK approach. In addition, we seek to pioneer the methodological development and clinical implementation of TDM-driven administration of JAKIs in multiple inflammatory states, collecting clinical responses to establish therapeutic intervals with pragmatic recommendations.

## Methods

### Study Participants

Adult patients (aged ≥18 years) capable of making informed decisions are eligible if they are receiving or about to receive JAKIs for the control of their inflammatory disease. This study includes currently commercialized JAKIs in Switzerland (abrocitinib, baricitinib, fedratinib, ruxolitinib, tofacitinib, and upadacitinib). Participants who wish to withdraw consent at any stage of the study, are incapable of judgment, or are under tutelage are excluded from this study.

### Ethical Considerations

The present protocol was approved by the Cantonal Research Ethics Committee of Vaud and transferred to the Ethics Committee of Northwestern and Central Switzerland, Cantonal Research Ethics Committee of Geneva, Cantonal Ethics Committee of Zurich, Ethics Committee of Eastern Switzerland, and Cantonal Ethics Committee of Bern in August 2023 (2023-00904). This research project is conducted in compliance with the protocol, the Declaration of Helsinki [56], the principles of Good Clinical Practice, the Human Research Ordinance [57], and the Human Research Act [58], as well as other locally relevant regulations. Patients are included in the study after signing an informed consent or a general consent. All data are handled in accordance with data protection laws, with participants’ identities coded and accessible only to authorized study staff to ensure strict confidentiality throughout the study. Participants undergoing detailed pharmacokinetics substudy investigations receive compensation for their time.

### Study Design

**Figure 1:**
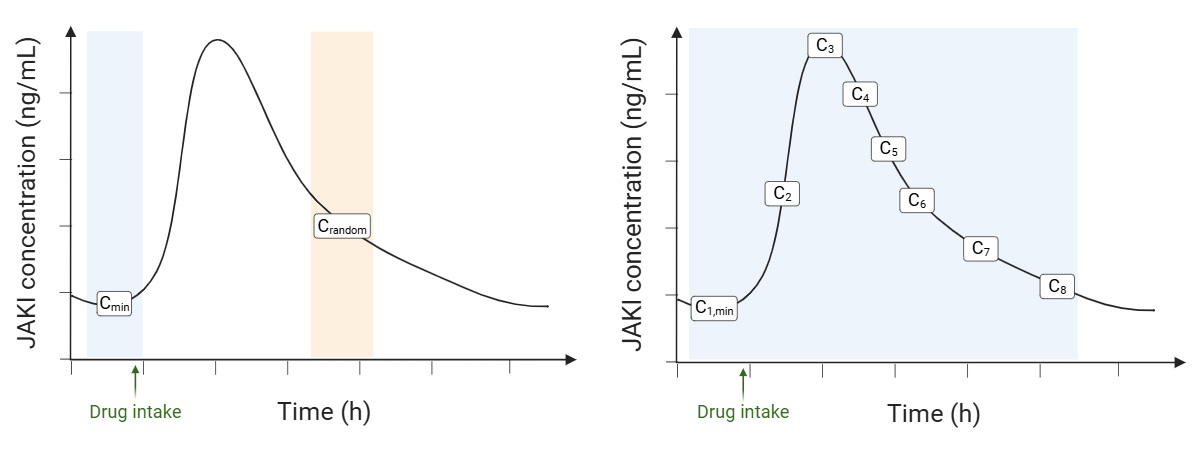
Study design. The study is divided into 2 parts: (A) the sparse sampling, where 1 blood sample can be collected during routine medical visits, and (B) the detailed pharmacokinetics substudy, where 8 blood samples are collected over a period of 8 hours. Cmin: samples collected at trough concentration; Crandom: samples collected at unselected time points over the dosing interval; C1,min: first samples collected at trough concentration; C2 to C7: samples collected at selected time points during the detailed pharmacokinetics substudy; JAKI: Janus kinase inhibitor. This figure was created using BioRender. Tachet, J [59].

This is a prospective observational study conducted in a real-world setting. As presented in Figure 1 [59], this project is divided in 2 main parts: sparse sampling and a detailed pharmacokinetics substudy. Sparse sampling is performed exclusively during routine medical visits, where 1 plasma sample is taken. This part of the study aims to obtain 1 to 3 trough plasma concentration samples (sample collected just before the next dose) and 1 to 3 random samples at unselected times over the entire dosing interval per patient. The detailed pharmacokinetics substudy is performed on specific patients in the Service of Clinical Pharmacology at the University Hospital of Lausanne to enrich the data collected during routine medical visits. It consists of collecting 1 sample before and 7 samples after taking a dose of JAKIs over an 8-hour period. This part of the study can be repeated for specific consenting patients in whom the introduction of an interacting drug raises the question of a DDI, or for those experiencing pathophysiological changes or acute intervention (eg, extrarenal epuration, gastric bypass, or plasmapheresis). In accordance with patient preferences and the study’s requirements, participants could either undergo a detailed pharmacokinetics investigation or provide additional sparse samples. Dosing results, including in cases of potential DDIs, are communicated to the treating physician for information purposes only, with any dose adjustment left to their discretion. In addition, pharmacogenetic analysis, which aims to identify genetic variants (ie, single-nucleotide polymorphisms) that affect the activity of drug-metabolizing enzymes or transporters and thereby influence overall drug exposure, will be performed in a subset of patients who provided separate consent for genetic testing. If clinicians consider phenotyping clinically relevant, a metabolic ratio analysis can provide additional medical insights related to JAKI metabolism [60]. The total study duration, including patient recruitment and the pharmacokinetics investigations, is estimated to last approximately 24 months.

### Source Data

The study data are recorded on a dedicated TDM report form and treated confidentially according to Swiss law data protection legislation. The actual times of the last drug intake preceding each sampling, along with the times of blood samplings themselves, are precisely recorded and documented. Specific information is also collected at each sampling time: body weight, height, JAKI (dose amount and administration frequency), diagnosis, comorbidities, list of comedications, clinical scores (eg, Disease Activity Score-28, Bath Ankylosing Spondylitis Disease Activity Index), and any information on organ dysfunction. ADRs reported by the patient during the investigation (Common Terminology Criteria for Adverse Events classification; version 5.0) are also documented through TDM report forms to capture the safety profile of the drugs being studied (Table 1). Other relevant clinical information (concomitant diseases, biomarkers) is extracted from the medical records and recorded with study data. Reasons for treatment interruption are documented in all cases. Adherence to treatment is assessed at the standard visit with physicians and nurses before filling out the TDM form. Information retrieved from each TDM report form and JAKI drug levels is entered into the Research Electronic Data Capture electronic case report form by a study team member. Before any pharmacokinetics or statistical analysis, the data entry is double-checked by the study coordinator.

**Table 1:** Data collected for the project.

Data type and subcategory			Unit
**General information**			
	Patient identification number (coded)	Category	
	Physician name	Category	
**Clinical justification**			
	Therapeutic monitoring not otherwise specified	Category	
	Unsatisfactory response and therapeutic failure	Category	
	Suspected drug interactions	Category	
	Dose adjustment for organ failure	Category	
	Suspected toxicity	Category	
**Diagnosis**			
	Indications for treatment with JAKIs^a^	Category	
	Comorbidities	Category	
**Clinical response**			
	Global clinical response	Value	
	DAS-28-ESR^b^	Value	
	DAS-28-CRP^c^	Value	
	ASDAS-ESR^d^	Value	
	BASDAI^e^	Value	
	BASFI^f^	Value	
	DAS68-ESR^g^	Value	
	DAS68-CRP^h^	Value	
	EASI^i^	Value	
	SCORAD^j^	Value	
	PGA scale^k^	Value	
	SELENA-SLEDAI^l^	Value	
	Harvey-Bradshaw	Value	
	Modified Truelove and Witts Severity Index	Value	
	Partial Mayo Score	Value	
	SSCAI^m^	Value	
	MPN 10^n^	Value	
**Organ dysfunction**			
	Heart failure	Category	
	Renal dysfunction	Category	
	Liver dysfunction	Category	
	Support (hemodialysis, CRRT^o^, and other)	Category	
**Sign of toxicity**			
	Sign of toxicity (CTCAE^p^, version 5.0)	Category	
**Body weight, height, and environmental factors**			
	Age (year of birth)	Date	
	Gender	Category	
	Ethnic background	Category	
	Body weight	kg	
	Height	cm	
	BMI	kg/m^2^	
	Smoking habits	Category	
	Alcohol consumption	Category	
**Laboratory values**			
	Creatinine	µmol/L	
	Erythrocyte	T/L	
	Hemoglobin	G/L	
	Neutrophile	G/L	
	Thrombocyte	G/L	
	Bilirubin	µmol/L	
	ALT^q^	U/L	
	AST^r^	U/L	
	Inflammatory biomarkers^s^	—^t^	
**Drug**			
	Molecule measured (ATC^u^ code)	Category	
	Dosage	mg	
	Number of daily intakes	Value	
	Level of therapeutic adherence	Percentage	
**Blood sampling and last given dose**			
	Date and time of blood sampling	Date and time	
	Date and time of the last dose	Date and time	
	Date of initiation or last change of dosing	Date	
	Time after dose	Time	
**Comedications**			
	Comedication (ATC code)	Category	
**Concentration measured**			
	Concentration measured	ng/mL	

^a^JAKI: Janus kinase inhibitor.

^b^DAS-28-ESR: Disease Activity Score-28 for rheumatoid arthritis with erythrocyte sedimentation rate.

^c^DAS-28-CRP: Disease Activity Score-28 for rheumatoid arthritis with C-reactive protein.

^d^ASDAS-ESR: Axial Spondyloarthritis Disease Activity Score with erythrocyte sedimentation rate.

^e^BASDAI: Bath Ankylosing Spondylitis Disease Activity Index.

^f^BASFI: Bath Ankylosing Spondylitis Functional Index.

^g^DAS-68-ESR: Disease Activity Score-68 for rheumatoid arthritis with erythrocyte sedimentation rate.

^h^DAS-68-CRP: Disease Activity Score-68 for rheumatoid arthritis with C-reactive protein.

^i^EASI: Eczema Area and Severity Index.

^j^SCORAD: Scoring Atopic Dermatitis.

^k^PGA: Physician Global Assessment.

^l^SELENA-SLEDAI: Safety of Estrogens in Lupus Erythematosus National Assessment-Systemic Lupus Erythematosus Disease Activity Index.

^m^SCCAI: Simple Clinical Colitis Activity Index.

^n^MPN-10: Myeloproliferative Neoplasm Symptom Assessment Form.

^o^CRRT: continuous renal replacement therapy.

^p^CTCAE: Common Terminology Criteria for Adverse Events.

^q^ALT: alanine aminotransferase.

^r^AST: aspartate aminotransferase.

^s^Inflammatory biomarkers, for example, C-reactive protein and erythrocyte sedimentation rate.

^t^Units for inflammatory biomarkers depend on the specific markers selected.

^u^ATC: Anatomical Therapeutic Chemical classification.

### Drug-Level Measurements

Measurements of total plasma levels of JAKIs are performed by multiplex high-performance liquid chromatography coupled to tandem mass spectrometry as a part of the routine TDM service provided by the Laboratory of Clinical Pharmacology (University Hospital of Lausanne, Switzerland). A multiplex assay was developed and validated according to the French Society of Pharmaceutical Sciences and Techniques and International Council for Harmonisation of Technical Requirements for Pharmaceuticals for Human Use–M10 guidelines (European Medicine Agency) for the simultaneous quantification of JAKIs in plasma (abrocitinib, baricitinib, fedratinib, ruxolitinib, tofacitinib, and upadacitinib). The method was validated over the clinically relevant concentration ranges (0.5-200 ng/mL for abrocitinib, baricitinib, and upadacitinib; 0.5-400 ng/mL for ruxolitinib; 1-400 ng/mL for tofacitinib; and 10-800 ng/mL for fedratinib) [61].

In-house studies indicate that abrocitinib, baricitinib, tofacitinib, and upadacitinib are stable in whole blood at room temperature up to 96 hours, whereas ruxolitinib and fedratinib are stable in plasma (up to 96 hours at room temperature). Whole blood and plasma samples, together with the TDM report form, are shipped at room temperature in a plastic transport protection coffer to the Laboratory of Clinical Pharmacology. Whole blood samples are then immediately centrifuged (2000 g, 10 min, +4°C). The separated plasma is frozen at −80°C until analysis by high-performance liquid chromatography coupled to tandem mass spectrometry.

### Pharmacokinetic and Pharmacodynamic Modeling

The comprehensive population-based modeling and simulation of JAKI pharmacokinetics profile rely on nonlinear mixed effect modeling techniques, such as those implemented in the NONMEM (version 7.6.0; ICON Development Solutions) and Monolix (version 2024R1, Lixoft) software. PopPK approaches will enable the characterization of the average pharmacokinetics profile of JAKIs, including absorption, distribution, metabolism, and excretion, based on plasma concentrations and data pooled over all sampled individuals. It also allows for the quantification of inter and intraindividual variability and for the identification of various factors (covariates) contributing to this variability within the population.

For each JAKI, a classical stepwise procedure will be conducted to identify the popPK model that best fits the concentrations [62]. Models with several compartments, different absorption processes, and linear elimination will be compared. Interindividual variability will be sequentially tested on all the parameters, assumed to follow a log-normal distribution. Intraindividual variability will also be assessed. Covariates, such as body weight, age, sex, BMI, genetic polymorphisms, and comedication (eg, CYP and transporter inhibitors or inducers), susceptible to interact with JAKI metabolism will be tested for significance on the base model parameters using appropriate mathematical functions. DDIs will be identified based on concomitant medications reported in TDM report forms using the UpToDate drug interactions tool [63]. Interacting drugs will be classified as weak, moderate, or strong CYP or transporter inhibitors or inducers. As chronic inflammation in autoimmune diseases may alter drug pharmacokinetics, disease type (eg, GvHD, rheumatoid arthritis) and level of inflammation assessed with inflammatory biomarkers (eg, C-reactive protein) will also be tested for significance in the popPK model. Covariates will be sequentially tested using forward selection and backward elimination steps. The first phase involves adding each covariate to the base model, followed by combining the significant ones in multivariate analyses to establish an intermediate model. The second step is the one-by-one removal of covariates from the intermediate model to retain only the most significant ones. Significance levels of .05 and .01 will be used to statistically discriminate hierarchical models during the base model building and covariate forward insertion steps, and the backward elimination phase, respectively. Akaike information criterion will serve to discriminate nonnested models. Diagnostic plots and the relative SE will support model selection. Sensitivity analyses will be conducted to assess the robustness of our model by removing outlier patients with extreme parameter estimates or covariates, such as patients receiving CYP-modulating drugs or those with severe disease presentations. Moreover, well-established internal validation methods (prediction-corrected visual predictive checks and bootstrap) will be used to evaluate final model predictive performance and reliability [64-66]. If enough data are collected, cross-validation will be performed with repeated data-splitting, creating random subsets of the dataset, allocating 80% of the data for modeling and 20% for validation.

Treatment efficacy and safety will be ascertained by studying the correlation between drug levels and the occurrence of inadequate disease control and ADRs within the study population. Efficacy-related pharmacodynamics data are collected through clinical outcome scores, while data on ADRs and predictive biomarkers for response, nonresponse, or toxicity (eg, platelet, erythrocyte, hemoglobin, and neutrophil counts) are gathered in parallel (Table 1). Pharmacokinetics and exposure-response or toxicity models will be developed using popPK software. This will enable comprehensive pharmacokinetics and pharmacodynamic analyses for the formal establishment of target trough concentrations or other relevant pharmacokinetics parameters. Findings will be compared with the available literature to identify suitable concentration targets associated with an optimal efficacy and toxicity ratio.

### Statistical Considerations

The precision of model estimates depends on both the number of available samples and their distributions across the dosing interval. Data collected throughout the administration interval enhances the ability to develop an adequate model. Drugs with highly variable and complex pharmacokinetics require more samples for an accurate description [67]. Moreover, the identification of covariate effects relies on their distribution and variability within the population of interest. Preliminary popPK analyses showed that abrocitinib, baricitinib, fedratinib, ruxolitinib, and upadacitinib were best described by a 2-compartment model [47,50,68-72], while tofactinib was best described by a 1-compartment model [73,74]. Actually, simulation studies have established that the minimum sample size required for a popPK analysis based on a 2-compartment model to estimate the 95% CI around typical pharmacokinetics parameters, such as clearance and distribution volume within a 50% precision level with a power of 0.8, is at least 50 patients, provided that the study applies an appropriate sampling schedule [75]. More specifically, assuming a 30% intraindividual variability and 50% interindividual variability in clearance, 182 patients would theoretically be needed to demonstrate a 20% difference by adding a covariate, again with due consideration to the sampling schedule [76]. Because the model will be based on rich data from detailed pharmacokinetics substudy, a smaller sample size of at least 50 patients per drugs was considered sufficient to establish popPK parameter values and coefficients for clinically significant covariates with a sufficient degree of precision, as it has already been the case in previous popPK studies that were conducted in similar patients [77-80].

## Results

The patient recruitment began in August 2023, following approval from the Ethics Committee. As of August 2024, a total of 107 patients had been recruited and 276 plasma samples analyzed. Most patients recruited were taking ruxolitinib (n=44, 41.1%), upadacitinib (n=39, 36.4%), and baricitinib (n=11, 10.3%). Of these, 23 (21.5%) participants taking either ruxolitinib (n=10, 43%), upadacitinib (n=10, 43%), or baricitinib (n=3, 13%) were enrolled in the detailed pharmacokinetics substudy over a period of 7 to 10 hours. Table 2 presents the overall and drug-specific population characteristics. Patient population was predominantly female individuals (62/107, 57.9%), with a median age of 51 (17-87) years and a median body weight of 69 (39-132) kg.

**Table 2:** Summary of characteristics of the patient population (data were extracted in August 2024).

					Abrocitinib		Baricitinib		Fedratinib		Ruxolitinib		Tofacitinib		Upadacitinib		Total
**Summary of characteristics of patients included in the study, n (%)**																	
	Patients			2 (1.8)		11 (10.3)		1 (0.9)		44 (41.1)		10 (9.3)		39 (36.4)		107	
	Samples			3 (1.1)		36 (13)		1 (0.4)		118 (42.8)		11 (4)		107 (38.8)		276	
**Demographic characteristics**																	
	Age (y), median (range)			38 (29-47)		62 (17-83)		76 (76)		64 (23-87)		53 (25-74)		47 (19-87)		51 (17-87)	
	**Sex, n (%)**																
			Male	0 (0)		3 (27.3)		1 (100)		25 (56.8)		3 (30)		13 (33.3)		45 (42.1)	
			Female	2 (100)		8 (72.7)		0 (0)		19 (43.2)		7 (70)		26 (66.7)		62 (57.9)	
**Anthropometric characteristics, median (range)**																	
	Body weight (kg)			58 (55-60)		70 (47-82)		85 (85)		67 (40-112)		72.3 (46-100)		73 (49-132)		69 (39-132)	
	Height (cm)			161 (160-162)		165 (152-178)		175 (175)		172 (146-202)		162 (150-190)		165 (157-188)		167 (146-202)	
	BMI (kg/m^2^)			22 (21-23)		26 (16-35)		28 (28)		24 (15-32)		27 (20-34)		26 (18-43)		25 (15-49)	
**Clinical characteristics**																	
	**Pathologies, n (%)**																
		Atopic dermatitis		2 (100)		0 (0)		0 (0)		0 (0)		0 (0)		3 (8)		5 (4.7)	
		Vitiligo		0 (0)		0 (0)		0 (0)		0 (0)		0 (0)		1 (3)		1 (0.9)	
		Dermatomyositis		0 (0)		1 (9)		0 (0)		0 (0)		1 (10)		1 (3)		3 (2.8)	
		Lichen planus		0 (0)		2 (18)		0 (0)		0 (0)		0 (0)		0 (0)		2 (1.9)	
		Eczema		0 (0)		1 (9)		0 (0)		0 (0)		0 (0)		0 (0)		1 (0.9)	
		Eczema herpeticum		1 (50)		0 (0)		0 (0)		0 (0)		0 (0)		0 (0)		1 (0.9)	
		Alopecia totalis		0 (0)		1 (9)		0 (0)		0 (0)		0 (0)		1 (3)		2 (1.9)	
		Hidradenitis suppurativa		0 (0)		0 (0)		0 (0)		0 (0)		0 (0)		2 (5)		2 (1.9)	
		Prurigo nodularis		0 (0)		0 (0)		0 (0)		0 (0)		0 (0)		1 (3)		1 (0.9)	
		Rheumatoid arthritis		0 (0)		6 (55)		0 (0)		0 (0)		2 (20)		3 (8)		11 (10.3)	
		Psoriatic arthritis		0 (0)		0 (0)		0 (0)		0 (0)		2 (20)		5 (13)		7 (6.5)	
		Axial spondyloarthritis		0 (0)		0 (0)		0 (0)		0 (0)		0 (0)		11 (28)		11 (10.3)	
		Juvenile idiopathic arthritis		0 (0)		0 (0)		0 (0)		0 (0)		1 (10)		0 (0)		1 (0.9)	
		Myelofibrosis		0 (0)		0 (0)		1 (100)		10 (23)		1 (10)		0 (0)		12 (11.2)	
		GvHD^a^		0 (0)		0 (0)		0 (0)		26 (59)		0 (0)		0 (0)		26 (24.3)	
		Polycythemia vera		0 (0)		0 (0)		0 (0)		3 (7)		0 (0)		0 (0)		3 (2.8)	
		Essential thrombocytopenia		0 (0)		0 (0)		0 (0)		2 (5)		0 (0)		0 (0)		2 (1.9)	
		VEXAS^b^ syndrome		0 (0)		0 (0)		0 (0)		2 (5)		0 (0)		0 (0)		2 (1.9)	
		Sarcoidosis		0 (0)		0 (0)		0 (0)		0 (0)		1 (10)		1 (3)		2 (1.9)	
		APECED^c^ syndrome		0 (0)		0 (0)		0 (0)		1 (2)		0 (0)		0 (0)		1 (0.9)	
		Ulcerative colitis		0 (0)		0 (0)		0 (0)		0 (0)		4 (40)		8 (21)		12 (11.2)	
		Crohn disease		0 (0)		0 (0)		0 (0)		0 (0)		0 (0)		11 (28)		11 (10.3)	
	**Number of comedications, n (%)**																
		0-4		2 (100)		6 (54.5)		0 (0)		22 (50)		7 (70)		27 (69.2)		64 (59.8)	
		5-8		0 (0)		5 (45.5)		0 (0)		13 (29.5)		3 (30)		8 (20.5)		29 (27.1)	
		9-12		0 (0)		0 (0)		1 (100)		9 (20.5)		0 (0)		4 (10.3)		14 (13.1)	

^a^GvHD: graft-versus-host disease.

^b^VEXAS syndrome: vacuoles, E1 enzyme, X-linked, autoinflammatory, somatic syndrome.

^c^APECED syndrome: autoimmune polyendocrinopathy-candidiasis-ectodermal dystrophy syndrome.

Ruxolitinib was primarily prescribed for GvHD (n=26, 59%) and myelofibrosis (n=10, 23%), while upadacitinib was mainly used for IBD (n=19, 49%) and rheumatological conditions (n=19, 49%). In total, 3 patients were treated with ruxolitinib for off-label conditions, such as autoimmune polyendocrinopathy-candidiasis-ectodermal dystrophy syndrome (n=1, 2%), or vacuoles, E1 enzyme, X-linked, autoinflammatory, somatic syndrome (n=2, 5%).

Most patients were administered upadacitinib at a dosage of 15 mg once daily and ruxolitinib at 10 mg twice daily (BID). One patient receiving ruxolitinib 10 mg BID exhibited unexpectedly high plasma exposure during the detailed pharmacokinetics substudy.

Inadequate response or treatment failure was reported in 14 (13.1%) of 107 patients. ADRs were present in 17 (38%) and 13 (33%) participants taking ruxolitinib and upadacitinib, respectively. The most frequent ADRs concerned the blood and lymphatic system (anemia, cytopenia, neutropenia, or thrombocytopenia), abnormal laboratory values (transaminases and cholesterol), and dermatological disorders (acne), which accounted for 30% (23/77), 16% (12/77), and 14% (11/77) of the total reported ADRs.

## Discussion

### Anticipated Findings

This protocol aims to monitor and individualize JAKI concentrations in the treatment of various inflammatory and hematological diseases. To the best of our knowledge, this is the first large-scale study to investigate the pharmacokinetics and pharmacodynamic profile of JAKIs in a real-world population and to develop method for monitoring them.

To date, most participants have been administered ruxolitinib, upadacitinib, or baricitinib, whereas only a limited proportion of participants received abrocitinib, fedratinib, and tofacitinib, reflecting their less frequent use in the study population. One individual diagnosed with GvHD and treated with 10 mg BID of ruxolitinib exhibited elevated concentrations and low elimination over time. Genotyping indicated a normal metabolizer phenotype, whereas phenotyping results revealed a phenoconversion with a reduced activity of the CYP2C19 and CYP3A, the latter likely due to environmental factors. The low activity could explain the abnormally elevated plasma drug concentrations. This patient notably experienced a viral respiratory infection requiring hospitalization shortly before inclusion in this study and was diagnosed with nonmelanoma skin cancer (basal cell carcinoma), documented in the literature as a ruxolitinib-related ADR [20,81-85].

During the first 12 months of inclusion, most patients receiving JAKIs demonstrated an adequate response to their treatment. However, some patients on ruxolitinib mainly experienced hepatic and hematological disorders ranging from mild to severe, while those on upadacitinib primarily reported dermatological ADRs. Dosages were frequently adjusted empirically over time by clinicians to manage toxicity and improve efficacy.

However, there are gaps in knowledge with regard to dosage and administration schemes for nonstandard patients. Significant interindividual variability has been reported for ruxolitinib in the literature [43,55], which may complicate the management of MPN and GvHD, potentially resulting in toxicities or reduced efficacy for some patients. According to published data, drug exposure was also influenced by the type of disease and comedications [43,50]. Current practices are based on drug manufacturers’ recommendations, often complemented by empirical decisions that account for patient characteristics, comorbidities, comedications, disease severity, and treatment response. For instance, upadacitinib has been used for a growing range of indications, from rheumatic (rheumatoid arthritis, psoriatic arthritis, and spondyloarthritis) and dermatological (atopic dermatitis, vitiligo, and alopecia) conditions to highly inflammatory diseases such as IBD, which require induction doses up to 3 times higher before transitioning to a maintenance phase [86]. Nonetheless, these approaches have so far been tested in the strict frame of clinical trials, which do not account for the complex real-world situation of many patients. In such cases, our understanding of JAKIs’ pharmacokinetics profiles and their impact on chronic disease management remains limited.

A wide range of clinical situations reported by physicians revealed unmet needs regarding the potential interest of TDM for JAKIs. A precision medicine approach, which can be assisted by the Tucuxi program [87,88], could thus help individualize treatments and improve clinical outcomes. JAKI monitoring represents a promising approach to maintain drug concentrations within narrow therapeutic indexes to overcome efficacy and safety concerns, while possibly addressing compliance issues as well. It represents a novel approach to provide an unambiguous benefit for patients and clinicians.

### Perspective

On the basis of the gathered pharmacokinetics data, the first popPK models for ruxolitinib and upadacitinib are expected to be developed in early 2025. By that time, the number of recruited patients should be sufficient for this analysis, which is expected to take several months. As a secondary end point, a pharmacokinetics and pharmacodynamic analysis will be conducted using the collected pharmacodynamics data to explore the relationships between drug concentration-response and toxicity. The results will be compared with existing literature to identify an optimal efficacy-toxicity ratio.

### Strengths and Limitations

Our patient population sample differs from the one observed in clinical trials due to the demographic characteristics and will enable us to perform robust analyses of pharmacokinetics variability in real-world settings. However, several limitations of the present protocol should be acknowledged. First, to ensure ethical compliance, participants incapable of judgment or who were under tutelage were excluded from the study, which may limit the generalizability of the findings. However, based on feasibility analyses, this group represents only a small proportion of patients eligible for JAKI treatment. Our cohort includes patients who are polymedicated with complex clinical situations, such as multimorbidity and altered metabolism, which likely contribute to the pharmacokinetics variability observed in real-world settings.

Furthermore, this study allows for the collection of ADRs not only during routine consultations but also during hospitalizations, when some ADRs may be severe. However, collecting pharmacodynamic data remains challenging. ADRs may be underreported in this study for several reasons. Outpatients typically report ADRs only during routine medical visits, which means that important information that may arise between consultations may be missed. This limitation is exacerbated by the infrequency of these visits, as patients may experience ADRs without the opportunity to communicate them. Health care professionals may also not systematically document reported ADRs due to time constraints. Besides, all clinical data and laboratory values are not always accessible, as patients are followed in different hospitals and cabinets across Switzerland. Finally, some clinical outcomes might also be underreported in our study, as they are not systematically assessed during routine clinical visits.

### Conclusions

This protocol aims to bring an original contribution to the monitoring of JAKIs by investigating the characteristics of their pharmacokinetics profile in real-world patients. It addresses an integrated strategy of treatment monitoring of JAKIs, based on relevant demographic and clinical factors, and measurement of circulating blood concentrations. The TDM of JAKIs appears instrumental in streamlining targeted immunotherapy as second- or third-line treatments. In the growing movement toward precision medicine, this novel research initiative is expected to improve the efficacy, effectiveness, tolerability, and long-term safety of JAKIs, and address DDIs and pharmacogenetic issues associated with them as prototypic medications with a narrow therapeutic index.

## Abbreviations

ADR: adverse drug reaction
BID: twice daily
CYP: cytochrome P450
DDI: drug-drug interaction
GvHD: graft-versus-host disease
IBD: inflammatory bowel disease
JAK: Janus kinase
JAKI: Janus kinase inhibitor
MPN: myeloproliferative neoplasm
popPK: population pharmacokinetics
STAT: signal transducer and activator of transcription
TDM: therapeutic drug monitoring

## Data Availability

The datasets generated or analyzed during this study are available from the corresponding author on reasonable request.
